# Real-world experience with cefepime/zidebactam under compassionate use

**DOI:** 10.1093/jac/dkag324

**Published:** 2026-11-10

**Authors:** Dilip K Dubey, Subramanian Swaminathan, Praveen K Tirlangi, Vishnu Rao Polati, Rajeev Soman, Rasika Sirsat, Abdul Ghafur, Raman Gaikwad, Vasant Nagvekar, Abi S Manesh, Irfana Mohammed, Harshad S Limaye, Pooja H Shah, Ankit Mittal, Pratik Y Patil, Nitin Gupta, Umang Agrawal, Manish Soneja, Sowmya Sridharan, Surabhi Madan, Sandeep K Mitra, Monalisa Sahu, Ayesha J Sunavala, Kedar K Toraskar, Tanu Singhal, Ashwini R Tayade, Neha Gupta, Anjely P Sebastian, Vijil Rahulan, Suneetha Narreddy, Erlinda R Ulloa, William R Miller, Vidya Krishna, Honey S Savla, Radhika N Sarda, Nitin Bansal, Shalmali B Inamdar, Harshith B Kadnur, Siddhant Shetty, Vikas Deswal, Elisabeth E Adderson, Samuel L Aitken, David van Duin, Timothy William, Andrew Shorr, Balaji Veeraraghavan, Thomas P Lodise

**Affiliations:** Institute of Critical Care Medicine, Medanta Super Speciality Hospital, Lucknow, Uttar Pradesh 226030, India; Department of Infectious Diseases, Gleneagles Global Hospitals, Chennai, Tamil Nadu 600100, India; Department of Infectious Diseases, Kasturba Medical College, Manipal Academy of Higher Education, Manipal, Udupi, Karnataka 576104, India; Department of Infectious Diseases, Apollo Hospital, Jubilee Hills, Hyderabad, Telangana State 500096, India; Department of Infectious Diseases, Jupiter Hospital, Pune, Maharashtra 411045, India; Department of Infectious Diseases, P.D. Hinduja National Hospital & Medical Research Centre, Mumbai, Maharashtra 400052, India; Department of Infectious Diseases, P.D. Hinduja National Hospital & Medical Research Centre, Mumbai, Maharashtra 400052, India; Department of Infectious Diseases, Apollo Hospitals, Chennai, Tamil Nadu 600006, India; Department of Infectious Diseases, Sahyadri Hospital, Pune, Maharashtra 411004, India; Department of Infectious Diseases, Lilavati Hospital and Research Centre, Mumbai, Maharashtra 400050, India; Department of Infectious Diseases Training and Research Centre, Christian Medical College Vellore, Vellore, Tamil Nadu 632004, India; Department of Infectious Diseases, KIMS - Sunshine Hospitals, Hyderabad, Telangana State 500003, India; Department of Infectious Diseases & Internal Medicine, Nanavati Max Super Speciality Hospital, Mumbai, Maharshtra 400056, India; Medical Oncology, Tata Memorial Hospital, Mumbai, Maharashtra 400012, India; Department of Infectious Diseases, AIG Hospitals, Hyderabad, Telangana State 500032, India; Department of Infectious Diseases, Krishna Institute of Medical Sciences, Secunderabad, Telangana State 500003, India; Department of Infectious Diseases, Kasturba Medical College, Manipal Academy of Higher Education, Manipal, Udupi, Karnataka 576104, India; Department of Infectious Diseases, P.D. Hinduja National Hospital & Medical Research Centre, Mumbai, Maharashtra 400052, India; Department of Medicine, All India Institute of Medical Sciences, New Delhi 110019, India; Department of Infectious Diseases, Kauvery Hospitals, Chennai 600 004, India; Infectious Diseases, Marengo CIMS Hospital, Science City, Sola, Ahmedabad, Gujarat 380060, India; Institute of Critical Care Medicine, Medanta Super Speciality Hospital, Lucknow, Uttar Pradesh 226030, India; Department of Infectious Diseases, Yashoda Hospitals, Hyderabad, Telangana State 500080, India; Department of Infectious Diseases, P.D. Hinduja National Hospital & Medical Research Centre, Mumbai, Maharashtra 400052, India; Department of Critical Care & Pulmonology, Wockhardt Hospital, Mumbai 400008, India; Department of Infectious Diseases, Kokilaben Dhirubhai Ambani Hospital and Medical Research Institute, Mumbai, Maharashtra 400053, India; Infectious Diseases and Adult Vaccination Clinic, Ishra Health, Nagpur 440010, Maharashtra, India; Infectious Diseases, NG Swastha & Fortis Memorial Research Institute, Gurugram, Haryana 122004, India; Department of Infectious Diseases, Kasturba Medical College, Manipal Academy of Higher Education, Manipal, Udupi, Karnataka 576104, India; Department of Heart and Lung Transplant, Krishna Institute of Medical Sciences, Secunderabad, Telangana State 500003, India; Department of Infectious Diseases, Apollo Hospital, Jubilee Hills, Hyderabad, Telangana State 500096, India; Department of Pediatrics, University of California Irvine School of Medicine, Irvine, CA 92617, USA; Division of Infectious Diseases, Children’s Hospital of Orange County, Orange, CA 92868, USA; Internal Medicine, Division of Infectious Diseases, Houston Methodist Hospital, Houston, TX 77030, USA; Department of Infectious Diseases, Apollo Hospitals, Chennai, Tamil Nadu 600006, India; Department of Medicine, Wockhardt Hospital, Mumbai, Maharashtra 400011, India; Department of Infectious Diseases, Artemis Hospitals, Gurugram, Haryana 122018, India; Department of Infectious Diseases, Rajiv Gandhi Cancer Institute, Delhi 110085, India; Department of Infectious Diseases, Kokilaben Dhirubhai Ambani Hospital and Medical Research Institute, Mumbai, Maharashtra 400053, India; Department of Infectious Diseases, AIG Hospitals, Hyderabad, Telangana State 500032, India; Department of Infectious Diseases, S.L. Raheja Hospital – A Fortis Associate, Mahim, Mumbai, Maharashtra 400016, India; Department of Internal Medicine, Medanta - The Medicity, Gurugram, Haryana 122001, India; Department of Infectious Diseases, St. Jude Children’s Research Hospital, Memphis TN 38105, USA; Division of Infectious Diseases, University of Michigan Medical School, Ann Arbor, MI 48109, USA; Department of Pharmacy, University of Michigan College of Pharmacy, Ann Arbor, MI 48109, USA; Department of Pharmacy, Michigan Medicine, Ann Arbor, MI 48109, USA; Department of Medicine, Microbiology and Immunology, University of North Carolina, Chapel Hill, NC 27514, USA; Department of Medicine, Subang Jaya Medical Centre, Subang Jaya, Selangor 47500, Malaysia; Department of Pulmonary and Critical Care Medicine, Medstar Washington Hospital Center, Washington, DC 20010, USA; Department of Clinical Microbiology, Christian Medical College and Hospital, Vellore, Tamil Nadu 632004, India; Department of Pharmacy Practice, Albany College of Pharmacy and Health Sciences, Albany, NY 12208, USA

## Abstract

**Introduction:**

Cefepime/zidebactam is a β-lactam/β-lactam enhancer combination with activity against carbapenem-resistant (CR) Gram-negative pathogens, including metallo-β-lactamase (MBL)-producing organisms. Before regulatory approval, cefepime/zidebactam was available through a compassionate-use programme for patients with CR Gram-negative infections who had limited available treatment options.

**Methods:**

We conducted a retrospective, multi-country analysis of patients who received cefepime/zidebactam through compassionate use between 2022 and 2025. Data included demographics, comorbidities, acute complications, prior antibiotic exposure, infection characteristics, microbiology, treatment details, and investigator-assessed outcomes.

**Results:**

Eighty-five patients were included (median age 48 years; 57.6% in an intensive care unit at initiation). Primary infections included pneumonia/respiratory infection (30.6%), bone and joint infection (24.7%), and complicated urinary tract infection/pyelonephritis (14.1%). *Pseudomonas aeruginosa* was the predominant pathogen (77.6%), followed by *Klebsiella pneumoniae* (14.1%) and *Acinetobacter baumannii* (11.8%); infections involving at least two Gram-negative pathogens occurred in 10.6%. Among isolates with available genotypic data, MBLs were common and predominantly NDM. The cefepime/zidebactam minimum inhibitory concentration (MIC)_50_ and MIC_90_ were 8 and 32 mg/L, respectively. Investigator-assessed clinical success at the end of therapy was achieved in 88.2% of patients (95% CI, 79.7–93.5), and microbiological eradication in 89.4% (95% CI, 81.1–94.3); on-therapy mortality was 10.6% (95% CI, 5.7–18.9). Treatment-emergent adverse events occurred in five patients (5.9%, 95% CI, 2.5–13.0), with one discontinuation.

**Conclusions:**

In this uncontrolled compassionate-use case series, cefepime/zidebactam treatment was accompanied by high investigator-assessed clinical success and microbiological eradication in patients with severe infections caused by CR Gram-negative pathogens. These observations require confirmation in prospective controlled studies.

## Introduction

Cefepime/zidebactam is a β-lactam/β-lactam enhancer combination comprising the fourth-generation cephalosporin cefepime and zidebactam, a bicycloacyl-hydrazide diazabicyclooctane with a dual mechanism of action.^1–3^ In addition to inhibiting selected class A and class C serine β-lactamases, including CTX-M-type extended-spectrum β-lactamases, KPC enzymes, and AmpC β-lactamases, zidebactam binds penicillin-binding protein 2 (PBP-2) with high affinity across Gram-negative bacteria. This activity complements cefepime binding to PBP-3 and, in Enterobacterales, PBP-1a/1b.^4–8^ The complementary binding profile provides activity against multidrug-resistant *Pseudomonas aeruginosa*, *Acinetobacter baumannii*, and Enterobacterales, including metallo-β-lactamase (MBL)-producing organisms.^3,6,9^ Preclinical studies and animal infection models have supported further clinical development.^2,4,6,7,10–14^

Before regulatory approval, cefepime/zidebactam was made available through a compassionate-use programme in selected regions on a case-by-case basis for patients with infections caused by carbapenem-resistant Gram-negative pathogens who had failed available treatment options. This study describes that experience, including patient and infection characteristics, prior antibiotic exposure, microbiological and resistance-genotype profiles, treatment patterns, and investigator-assessed outcomes at the end of therapy.

## Methods

### Study design and setting

This retrospective, multi-country observational study included patients who received cefepime/zidebactam through compassionate use between August 2022 and December 2025. Compassionate use was implemented on a case-by-case basis rather than under a formal protocol. Treating physicians were advised that compassionate use could be considered only when the following conditions were met: infection with a carbapenem-resistant pathogen, failure of available treatment options, a request from the treating physician or the patient’s family, and approval from the relevant regional health authority. Wockhardt supplied cefepime/zidebactam once these criteria were confirmed and all applicable regulatory, institutional ethics, and informed-consent requirements had been completed. No prespecified disease-severity threshold was used; requests were based on the treating physician’s clinical assessment of the infection. For inclusion in this retrospective analysis, patients were also required to have received cefepime/zidebactam for at least 4 days. No additional exclusion criteria were applied.

### Treatment administration

Cefepime/zidebactam was administered intravenously as cefepime 2 g plus zidebactam 1 g every 8 h, generally as a 60-min intermittent infusion, with adjustment for renal function and dialysis status (Appendix S1). Patients with augmented renal clearance (creatinine clearance >120 mL/min) received 2 g/1 g every 6 h. Paediatric patients received weight-based dosing (cefepime 50 mg/kg plus zidebactam 25 mg/kg every 8 h). In patients receiving intermittent haemodialysis, cefepime/zidebactam was administered after dialysis. Concomitant antimicrobial therapy and supportive care were at the discretion of the treating physician.

### Patient data collection

Participating sites submitted anonymized case-level data using standardized templates following completion of therapy (Appendix S1). Demographic variables included age, sex, hospital, and country. Baseline clinical characteristics included ICU admission, comorbidities, and immunocompromised status. Acute complications occurring during the index infection episode, including sepsis, septic shock, multi-organ dysfunction syndrome, renal dysfunction, respiratory failure, and other organ-specific complications, were recorded separately from baseline comorbidities.

Infection characteristics included both a primary infection type and all observed infection types during the clinical episode. The primary infection type was assigned centrally from the treating physician’s reported diagnosis using a prespecified coding rule: the first infection listed was designated as primary unless bacteraemia was explicitly described as secondary to a defined source (e.g. bacteraemia with complicated intra-abdominal infection or cellulitis), in which case the source infection was designated as primary and bacteraemia as secondary. Primary infection types were mutually exclusive; all infection syndromes documented during the clinical episode were captured separately and could overlap within a patient. The predefined infection categories included pneumonia/respiratory infection (including empyema), bloodstream/endovascular infection, complicated urinary tract infection (UTI)/pyelonephritis, intra-abdominal infection, skin and soft tissue infection, bone and joint infection (including skull-base osteomyelitis and otitis externa with bone involvement), central nervous system (CNS) infection, and infective endocarditis. This was a retrospective central classification based on site-submitted diagnoses and was not independently adjudicated.

Microbiological data included pathogen identification, central-laboratory isolate characterization, and available resistance-mechanism data. Pathogen categories were defined at the patient level and were not mutually exclusive. For the primary analysis, polymicrobial Gram-negative infection was defined as the presence of at least two Gram-negative pathogens during the same clinical episode. Gram-positive and fungal co-pathogens were not systematically captured and therefore were not included in this definition.

Treatment variables included cefepime/zidebactam dosing strategy, prior antimicrobial exposure, and treatment duration. Concomitant antimicrobial therapy during cefepime/zidebactam treatment was not routinely captured. Clinical outcomes were assessed by the treating investigator at each site and were not independently adjudicated. Clinical success was defined as resolution or clinically meaningful improvement in infection-related signs and symptoms at the end of therapy. Microbiological eradication was defined as documented clearance on repeat culture or presumed clearance when repeat culture was not obtained and the clinical infection resolved. Indeterminate outcomes were classified as failures in the primary analyses. On-therapy mortality was defined as death during cefepime/zidebactam treatment. Thirty-day mortality, 90-day mortality, and post-treatment recurrence were not routinely collected. Safety outcomes included site-reported treatment-emergent adverse events and treatment discontinuations.

### Microbiological methods

Available viable clinical isolates recovered at participating sites were submitted to the Wockhardt central reference laboratory for antimicrobial susceptibility testing. Central-laboratory results were used for microbiological analyses when isolates were received and remained viable for testing. Susceptibility to cefepime/zidebactam and comparator agents was determined by reference broth microdilution according to CLSI M07 methods.^15^ Comparator agents included cefepime, meropenem, imipenem/relebactam, imipenem, ceftolozane/tazobactam, ceftazidime/avibactam, aztreonam/avibactam, amikacin, levofloxacin, and colistin. Minimum inhibitory concentration (MIC) values were reported as doubling dilutions, with values exceeding the upper assay limit reported as greater than the highest concentration tested (e.g. >64 or >128 mg/L). MIC range, MIC_50_, and MIC_90_ were summarized at the isolate level. When multiple isolates were available from a single patient, all isolates were included in isolate-level analyses. For patient-level MIC analyses, the highest observed cefepime/zidebactam MIC was used.

All viable isolates received by the central reference laboratory were considered for whole-genome sequencing. Sequencing depended on isolate receipt, recovery, viability, and completion of characterization rather than on sequencing capacity at individual sites. Resistance determinants were categorized as MBLs (NDM, VIM, IMP, DIM, and intrinsic L-1 where applicable), OXA-type β-lactamases, extended-spectrum and other β-lactamases (including CTX-M, SHV, TEM, GES, VEB, and PME), AmpC variants (including PDC, ADC, CMY, DHA, PAC, and intrinsic SRT), and non-enzymatic mechanisms. The latter included porin alterations (e.g. OprD and OmpK36), efflux systems (e.g. MexAB–OprM), PBP modifications, quinolone target mutations (*gyrA*, *parC*, and *parE*), aminoglycoside-modifying enzymes, and other chromosomally encoded resistance determinants. For each isolate, the numbers of distinct β-lactamase families and resistance-mechanism categories were tabulated. Additional binary variables identified isolates harbouring more than one β-lactamase family, more than one MBL, or more than one resistance-mechanism category.

### Statistical analysis

All analyses were descriptive. Continuous variables are reported as medians (range) and categorical variables as counts and percentages. Wilson score 95% CIs were calculated for the principal overall clinical and safety outcome estimates. Analyses were conducted at the patient or isolate level, as appropriate. Clinical and microbiological outcomes were summarized overall and stratified by primary infection type, pathogen category, cefepime/zidebactam MIC category (≤4, 8, 16, 32–64, and >64 mg/L), and resistance genotype. MIC-based analyses were restricted to patients with centrally tested isolates and used the highest observed cefepime/zidebactam MIC when multiple isolates were available. Prespecified subgroup analysis was conducted among patients with *P. aeruginosa* infection using the same MIC strata. Resistance-genotype analyses included MBL status, PBP-3 mutation status, and other resistance determinants. No formal hypothesis testing or adjusted comparative analysis was performed.

## Results

### Patient and infection characteristics

A total of 85 patients received cefepime/zidebactam through compassionate use. Median age was 48 years (range 1.25–87), 8 patients (9.4%) were paediatric, 78.8% were male, and 90.6% were treated in India (Table 1). At treatment initiation, 49 patients (57.6%) were receiving ICU care.

**Table 1.** Baseline characteristics, comorbidities, complications, prior antibiotic exposure, infection types, microbiology, treatment, and outcomes of patients receiving cefepime/zidebactam under compassionate use (*n* = 85)

Parameter	Value
**Demographics**	
Age, median (range), years	48 (1.25–87)
Paediatric patients (<18 years)	8 (9.4%)
Male sex	67 (78.8%)
Country	India: 77 (90.6%); USA: 6 (7.1%); Other: 2 (2.4%)
**Clinical status at initiation**	
ICU admission at initiation	49 (57.6%)
**Treatment characteristics** ^a^	
Standard dosing regimen	45 (52.9%)
Renal-adjusted dosing	39 (45.9%)
Haemodialysis-adjusted (subset of renal-adjusted)	2 of 39
Duration of therapy, median (range), days	21 (4–95)
**Prior antibiotic exposure** ^b^	
Patients with available prior antibiotic data	83/85 (97.6%)
Received ≥1 prior antibiotic	83/83 (100.0%)
Received ≥2 prior antibiotics	70/83 (84.3%)
Received ≥3 prior antibiotics	55/83 (66.3%)
Received ≥4 prior antibiotics	35/83 (42.2%)
Number of prior antibiotics, median (range)	3 (1–9)
Ceftazidime/Avibactam	41/83 (49.4%)
Aztreonam	38/83 (45.8%)
Meropenem	38/83 (45.8%)
Colistin	37/83 (44.6%)
Polymyxin B	35/83 (42.2%)
Fosfomycin	17/83 (20.5%)
Tigecycline	14/83 (16.9%)
Cefiderocol	9/83 (10.8%)
Piperacillin/Tazobactam	6/83 (7.2%)
Minocycline	5/83 (6.0%)
Levofloxacin	4/83 (4.8%)
Teicoplanin	4/83 (4.8%)
Cefoperazone/Sulbactam	4/83 (4.8%)
Sulbactam	4/83 (4.8%)
Amikacin	3/83 (3.6%)
Doripenem	2/83 (2.4%)
Imipenem/Relebactam	2/83 (2.4%)
Eravacycline	2/83 (2.4%)
Ceftriaxone/Sulbactam	2/83 (2.4%)
Ceftolozane/Tazobactam	2/83 (2.4%)
Linezolid	1/83 (1.2%)
Imipenem	1/83 (1.2%)
Ceftriaxone	1/83 (1.2%)
Ceftriaxone/sulbactam/disodium edetate	1/83 (1.2%)
Chloramphenicol	1/83 (1.2%)
Plazomicin	1/83 (1.2%)
Ceftazidime	1/83 (1.2%)
Ampicillin/Sulbactam	1/83 (1.2%)
Combination therapy	Not captured in dataset
**Comorbidities** ^c,d^	
Malignancy	11 (12.9%)
Solid organ transplant	13 (15.3%)
Other immunocompromised	6 (7.1%)
Immunocompromised/transplant-related (composite)	27 (31.8%)
Chronic lung disease	7 (8.2%)
Metabolic/endocrine disease	9 (10.6%)
Cardiovascular disease	15 (17.6%)
Neurologic condition	13 (15.3%)
Renal disease	7 (8.2%)
Other comorbidities	17 (20.0%)
Multiple comorbidities (≥2 categories)	27 (31.8%)
No comorbidities reported	14 (16.5%)
**Complications** ^c,e^	
Sepsis/septic shock/MODS	32 (37.6%)
Renal dysfunction (AKI/CKD/CRRT)	23 (27.1%)
Respiratory failure (ARDS/ECMO/mechanical ventilation)	14 (16.5%)
Intra-abdominal complications	5 (5.9%)
CNS complications	6 (7.1%)
Skin/soft tissue–related complications	5 (5.9%)
Bone/joint or surgical complications	4 (4.7%)
Other complications	3 (3.5%)
No major complications reported	26 (30.6%)
**Primary infection type** ^f^	
Pneumonia/respiratory	26 (30.6%)
Bone and joint infection	21 (24.7%)
UTI/pyelonephritis	12 (14.1%)
Intra-abdominal infection	9 (10.6%)
Skin and soft tissue infection	8 (9.4%)
CNS infection	4 (4.7%)
Bloodstream infection/endovascular	3 (3.5%)
Infective endocarditis	2 (2.4%)
**Observed infection types** ^c,g^	
Pneumonia/respiratory	30 (35.3%)
Bone and joint infection	22 (25.9%)
Complicated UTI/pyelonephritis	13 (15.3%)
Intra-abdominal infection	9 (10.6%)
Skin and soft tissue infection	9 (10.6%)
CNS infection	4 (4.7%)
Infective endocarditis	2 (2.4%)
Bloodstream/endovascular infection^h^	16 (18.8%)
**Microbiology** ^c^	
*Pseudomonas aeruginosa*	66 (77.6%)
*Klebsiella pneumoniae*	12 (14.1%)
*Acinetobacter baumannii*	10 (11.8%)
*Escherichia coli*	3 (3.5%)
Other Gram-negative pathogens	3 (3.5%)
Polymicrobial infection (≥2 GN)	9 (10.6%)
**Outcomes**	
Clinical success at end of therapy	75 (88.2%) [95% CI 79.7–93.5]
Microbiologic eradication at end of therapy	76 (89.4%) [95% CI 81.1–94.3]
On-therapy mortality	9 (10.6%) [95% CI 5.7–18.9]
**Safety** ^i^	
Treatment-emergent adverse events	5 (5.9%) [95% CI 2.5–13.0]
Discontinuation due to adverse events	1 (1.2%) [95% CI 0.2–6.4]

^a^Standard dosing was cefepime 2 g plus zidebactam 1 g intravenously every 8 h over 60 min. Renal-adjusted dosing included dose reduction, interval extension, or step-down dosing according to creatinine clearance. Haemodialysis-adjusted dosing was administered after haemodialysis and is included within the renal-adjusted group. Paediatric dosing was cefepime 50 mg/kg plus zidebactam 25 mg/kg every 8 h.

^b^Prior antibiotic exposure was available for 83 patients. Individual agents were not mutually exclusive. Concomitant antimicrobial therapy during cefepime/zidebactam treatment was not routinely captured.

^c^Comorbidities, complications, observed infection types, and pathogens were not mutually exclusive; percentages may exceed 100%.

^d^Comorbidities were present at baseline. The immunocompromised/transplant-related composite included malignancy, haematological or oncological disease, solid organ transplantation, and other immunocompromised states.

^e^Complications were acute events during the index infection episode and were recorded separately from baseline comorbidities. MODS, multi-organ dysfunction syndrome; AKI, acute kidney injury; ARDS, acute respiratory distress syndrome.

^f^Primary infection type was assigned centrally and was mutually exclusive.

^g^Observed infection types included all syndromes documented during the clinical episode and were not mutually exclusive. Ten patients had two infection types, and four had three or more.

^h^Bloodstream/endovascular infection in the observed-infection section denotes bacteraemia recorded as secondary to another primary infection.

^i^Treatment-emergent adverse events occurred in five patients and included renal dysfunction (*n* = 3), neurological events (*n* = 3), and elevated hepatic enzymes (*n* = 2). Cefepime/zidebactam was withdrawn in one patient after 21 days in the setting of kidney function decline and elevated hepatic enzymes.

Comorbidities were common, including immunocompromised, malignancy, or transplant-related conditions in 27 patients (31.8%). During the index infection episode, sepsis, septic shock, or multi-organ dysfunction syndrome occurred in 32 patients (37.6%), renal dysfunction in 23 (27.1%), and respiratory failure in 14 (16.5%). Cefepime/zidebactam was administered using standard dosing in 45 patients (52.9%) and renal-adjusted dosing in 39 (45.9%); median treatment duration was 21 days (range 4–95).

### Prior antibiotic exposure

Prior antibiotic exposure data were available for 83 patients. All had received at least one prior antibiotic, with 84.3% receiving ≥2 and 66.3% receiving ≥3 agents before cefepime/zidebactam initiation. The median number of prior antibiotics was 3 (range 1–9). The most common prior agents were ceftazidime/avibactam, aztreonam, meropenem, colistin, and polymyxin B (Table 1).

### Primary and observed infection types

Primary infections were heterogeneous, most commonly pneumonia or respiratory infection (30.6%), bone and joint infection (24.7%), and UTI/pyelonephritis (14.1%) (Table 1). Multiple infection syndromes were present in 14 patients (16.5%). *P. aeruginosa* was the predominant pathogen, identified in 66 patients (77.6%), followed by *Klebsiella pneumoniae* (14.1%) and *A. baumannii* (11.8%). Polymicrobial infections occurred in nine patients (10.6%) (Tables 1 and 2).

**Table 2.** Distribution of key pathogens by primary infection type (*n* = 85)

Infection type^a,b^	*n*	Number of patients with each pathogen,^c^ by primary infection type					
		*P. aeruginosa n* (%)	*K. pneumoniae n* (%)	*A. baumannii n* (%)	*E. coli* *n* (%)	Other GN^d^ *n* (%)	Polymicrobial *n* (%)^e^
Pneumonia	26	21 (80.8)	3 (11.5)	4 (15.4)	0 (0.0)	1 (3.8)	3 (11.5)
Bone and joint infection	21	18 (85.7)	1 (4.8)	2 (9.5)	0 (0.0)	1 (4.8)	1 (4.8)
Complicated UTI/pyelonephritis	12	9 (75.0)	2 (16.7)	0 (0.0)	0 (0.0)	1 (8.3)	0 (0.0)
Intra-abdominal infection	9	6 (66.7)	3 (33.3)	0 (0.0)	1 (11.1)	0 (0.0)	1 (11.1)
Skin and soft tissue infection	8	7 (87.5)	3 (37.5)	1 (12.5)	1 (12.5)	0 (0.0)	4 (50.0)
CNS infection	4	1 (25.0)	0 (0.0)	3 (75.0)	0 (0.0)	0 (0.0)	0 (0.0)
Bloodstream infection/endovascular	3	2 (66.7)	0 (0.0)	0 (0.0)	1 (33.3)	0 (0.0)	0 (0.0)
Infective endocarditis	2	2 (100.0)	0 (0.0)	0 (0.0)	0 (0.0)	0 (0.0)	0 (0.0)
Total	85	66 (77.6%)	12 (14.1%)	10 (11.8%)	3 (3.5%)	3 (3.5%)	9 (10.6%)

^a^Values are the number (%) of patients within each primary infection category with the specified pathogen.

^b^Primary infection type was assigned centrally and was mutually exclusive.

^c^Pathogen categories were not mutually exclusive; patients with polymicrobial infection contributed to more than one pathogen column.

^d^Other Gram-negative pathogens comprised *Serratia marcescens*, *Morganella morganii*, and *Stenotrophomonas maltophilia* (one patient each).

^e^Polymicrobial infection was defined as at least two Gram-negative pathogens identified during the same clinical episode.

### Antimicrobial susceptibility

Central laboratory susceptibility testing was available for 77 isolates from 72 patients. Cefepime/zidebactam demonstrated an MIC range of 0.12 to >64 mg/L, with an MIC_50_ of 8 mg/L and an MIC_90_ of 32 mg/L (Table 3). Overall, 67.5% and 87.0% of isolates had MICs ≤8 mg/L and ≤16 mg/L, respectively (Table S1, available as Supplementary data at *JAC* Online). Among *P. aeruginosa* isolates, the MIC_50_ and MIC_90_ were 8 and 16 mg/L, respectively, whereas higher MICs were observed among *A. baumannii* isolates (Table S2). For most comparator agents, MIC_50_ and MIC_90_ values exceeded 128 mg/L, with lower values observed for aztreonam/avibactam and colistin (Table 3).

**Table 3.** In vitro activity of cefepime/zidebactam and comparator agents against isolates tested at the central laboratory^a^

Agent	*n* ^b,c^	MIC range (mg/L)^d^	MIC_50_^e^	MIC_90_^e^
Cefepime–zidebactam	77	0.12 to >64	8	32
Cefepime	75	16 to >128	>128	>128
Meropenem	75	4 to >128	>128	>128
Imipenem	75	1 to >128	>128	>128
Imipenem–relebactam	75	0.25 to >128	>128	>128
Ceftolozane–tazobactam	75	4 to >128	>128	>128
Ceftazidime–avibactam	75	8 to >128	>128	>128
Aztreonam–avibactam	75	0.06 to >128	64	>128
Amikacin	75	0.06 to >128	>128	>128
Levofloxacin	75	2 to >128	128	>128
Colistin	75	0.06 to >128	0.5	8

^a^Susceptibility testing was performed on isolates received and tested at the central laboratory using reference broth microdilution methods.

^b^Multiple isolates per patient were included where available; the cefepime/zidebactam denominator (*n* = 77) reflects the total number of unique isolates tested rather than the number of patients.

^c^Denominators vary by antimicrobial agent because not all isolates had valid MIC results for every comparator; comparators were tested on a 75-isolate subset for which the full panel was completed.

^d^MIC range reflects the observed minimum and maximum MIC values for each agent. Values exceeding the upper assay limit are reported as >64 or >128 mg/L as appropriate.

^e^MIC_50_ and MIC_90_ represent the 50th and 90th percentiles of the observed MIC distribution.

### Resistance mechanisms

Genotypic resistance data were available for 73 isolates from 69 patients. MBLs were detected in 59 isolates (80.8%), predominantly NDM (64.4%), and were most common among *P. aeruginosa* isolates (Table 4). MBLs were also identified in *A. baumannii* (7/9, 77.8%), *K. pneumoniae* (5/7, 71.4%), and both *E. coli* isolates (2/2, 100%). At the patient level, 56 of 69 patients (81.2%) harboured at least one MBL-producing organism (Table S3). Additional resistance mechanisms were frequent, including OXA-type β-lactamases (90.4%), AmpC variants (84.9%), and non-enzymatic mechanisms such as porin alterations and efflux modifications (87.7%) (Table 4). Among MBL-positive isolates, 96.6% harboured at least one additional resistance mechanism, and 68 of 73 genotyped isolates (93.2%) carried more than one β-lactamase family (Table 4). The median number of β-lactamase families and resistance mechanism categories per isolate was 3 and 4, respectively. PBP-3 mutations were detected in 14 isolates (19.2%) and 14 patients (20.3%).

**Table 4.** Distribution of resistance mechanisms among isolates with available genotypic data (*n* = 73 isolates)^a,b,c^

Pathogen	*n*	MBL *n* (%)^d^	OXA-type *n* (%)^e^	ESBL/other *n* (%)^f^	AmpC variants *n* (%)^g^	Other resistance mechanisms *n* (%)^h,i^
*P. aeruginosa*	52	43 (82.7%)	48 (92.3%)	1 (1.9%)	49 (94.2%)	46 (88.5%)
*K. pneumoniae*	7	5 (71.4%)	6 (85.7%)	6 (85.7%)	1 (14.3%)	5 (71.4%)
*A. baumannii*	9	7 (77.8%)	9 (100.0%)	1 (11.1%)	9 (100.0%)	9 (100.0%)
*E. coli*	2	2 (100.0%)	2 (100.0%)	1 (50.0%)	1 (50.0%)	2 (100.0%)
Other Gram-negatives^j^	3	2 (66.7%)	1 (33.3%)	1 (33.3%)	2 (66.7%)	2 (66.7%)

^a^Analysis included 73 of 77 isolates received and identified at the Wockhardt central laboratory with available genotypic data. Four isolates (three *P. aeruginosa* and one *K. pneumoniae*) without completed genotypic characterization were excluded. Values are the number (%) of isolates within each pathogen group carrying each resistance category.

^b^Resistance categories were not mutually exclusive; isolates could harbour multiple mechanisms, and row percentages may exceed 100%.

^c^Percentages used the pathogen-specific number of isolates with available genotypic data as the denominator.

^d^MBLs included NDM, VIM, IMP, DIM, and the intrinsic L1 enzyme of *S. maltophilia*.

^e^OXA-type β-lactamases included carbapenem-hydrolyzing and narrow-spectrum OXA-family enzymes.

^f^ESBLs and other β-lactamases included CTX-M, SHV, TEM, GES, VEB, and PME families.

^g^AmpC variants included PDC in *P. aeruginosa*, ADC in *A. baumannii*, acquired CMY, DHA, and PAC enzymes in Enterobacterales, and intrinsic SRT enzymes in *S. marcescens*.

^h^Other resistance mechanisms included porin alterations, efflux-pump up-regulation, penicillin-binding protein modifications, quinolone target mutations, aminoglycoside-modifying enzymes, and other chromosomally encoded resistance determinants.

^i^PBP-3 modifications were detected in 14 of 73 isolates: 10 of 52 *P. aeruginosa*, 2 of 9 *A. baumannii*, 1 of 7 *K. pneumoniae*, and 1 of 2 *E. coli*. Eight of these isolates also harboured an MBL. The additional MBL-positive/PBP-3-positive patient in Table 8 reflects patient-level aggregation across isolates in a polymicrobial infection.

^j^Other Gram-negative organisms comprised *S. marcescens*, *M. morganii*, and *S. maltophilia* (one isolate each).

### Clinical outcomes

Overall, investigator-assessed clinical success at the end of therapy was achieved in 75 patients (88.2%; 95% CI 79.7–93.5), microbiological eradication in 76 (89.4%; 95% CI 81.1–94.3), and on-therapy mortality occurred in nine (10.6%; 95% CI 5.7–18.9) (Table 1). Outcomes varied according to baseline illness severity and host status. Clinical success was lower among patients receiving care in an intensive care unit at treatment initiation than among those who were not (81.6% versus 97.2%), among patients with sepsis-spectrum complications than among those without these complications (78.1% versus 94.3%), and among immunocompromised or transplant-related patients than among other patients (81.5% versus 91.4%). On-therapy mortality showed a similar pattern, occurring in 18.4% of patients receiving care in an intensive care unit at treatment initiation, 25.0% of those with sepsis-spectrum complications, and 18.5% of immunocompromised or transplant-related patients (Table S4).

Outcomes also varied by infection type (Table 5). Clinical success was lowest among patients with pneumonia or respiratory infection (73.1%), which also had the highest on-therapy mortality (26.9%). In contrast, outcomes were favourable among patients with bone and joint infection, UTI/pyelonephritis, and CNS infection. Outcomes were generally consistent across pathogen groups (Table 6). Clinical success was achieved in 86.4% of patients with *P. aeruginosa* infection, 100.0% with *K. pneumoniae*, and 90.0% with *A. baumannii*. Microbiologic eradication rates were similarly high across pathogen groups. Outcomes were similar between monomicrobial and polymicrobial infections.

**Table 5.** Clinical outcomes by primary infection type (*n* = 85)

Infection type^a^	*n*	Clinical success at end of therapy^b,c,d,e^ *n* (%)	Microbiologic eradication at end of therapy^b,c,e,f^ *n* (%)	On-therapy mortality *n* (%)^b,c,g^
Pneumonia	26	19 (73.1%)	22 (84.6%)	7 (26.9%)
Bone and joint infection	21	20 (95.2%)	20 (95.2%)	0 (0.0%)
Complicated UTI/pyelonephritis	12	12 (100.0%)	10 (83.3%)	1 (8.3%)
Intra-abdominal infection	9	8 (88.9%)	9 (100.0%)	0 (0.0%)
Skin and soft tissue infection	8	8 (100.0%)	7 (87.5%)	0 (0.0%)
CNS infection	4	4 (100.0%)	4 (100.0%)	0 (0.0%)
Bloodstream infection/endovascular	3	2 (66.7%)	2 (66.7%)	1 (33.3%)
Infective endocarditis	2	2 (100.0%)	2 (100.0%)	0 (0.0%)

^a^Primary infection types reflect the single centrally assigned primary infection type (mutually exclusive).

^b^Outcomes are reported at the patient level.

^c^Percentages are calculated using the number of patients within each primary infection type as the denominator.

^d^Clinical success: investigator-assessed resolution or clinically meaningful improvement in infection-related signs and symptoms at the end of cefepime/zidebactam therapy.

^e^Indeterminate outcomes were conservatively classified as failures for both clinical success and microbiologic eradication. Three patients had indeterminate microbiological outcomes (pneumonia, *n* = 1; complicated urinary tract infection/pyelonephritis, *n* = 1; bloodstream/endovascular infection, *n* = 1), and two patients had indeterminate clinical outcomes (both pneumonia).

^f^Microbiological eradication: investigator-assessed documented clearance on repeat culture or presumed clearance when repeat culture was not obtained and the clinical infection resolved.

^g^On-therapy mortality: death occurring during cefepime/zidebactam treatment, regardless of attribution.

**Table 6.** Clinical outcomes by pathogen, with mono- versus polymicrobial stratification

Pathogen^a,b^	*n* (total)	*n* mono	*n* poly	Clinical success *n* (%)^c^	Microbiologic eradication *n* (%)^c^	On-therapy mortality *n* (%)^c^
*P. aeruginosa*	66	58	8	57 (86.4%)	58 (87.9%)	8 (12.1%)
*K. pneumoniae*	12	7	5	12 (100.0%)	10 (83.3%)	0 (0.0%)
*A. baumannii*	10	6	4	9 (90.0%)	10 (100.0%)	1 (10.0%)
*E. coli*	3	3	0	3 (100.0%)	3 (100.0%)	0 (0.0%)
Other Gram-negatives^d^	3	2	1	3 (100.0%)	3 (100.0%)	0 (0.0%)
Monomicrobial infection (overall)^e^	76	—	—	66 (86.8%)	68 (89.5%)	9 (11.8%)
Polymicrobial infection (overall)^e^	9	—	—	9 (100.0%)	8 (88.9%)	0 (0.0%)

^a^Pathogen categories were defined at the patient level and were not mutually exclusive. Patients with polymicrobial infection contributed to each applicable pathogen category.

^b^‘n mono’ and ‘n poly’ denote patients with the specified pathogen alone or with at least one additional Gram-negative pathogen, respectively.

^c^Outcome definitions follow Table 5. Clinical success and microbiological eradication were investigator assessed, and indeterminate outcomes were classified as failures. Three patients had indeterminate microbiological outcomes, all with monomicrobial P. *aeruginosa* infection. Two patients had indeterminate clinical outcomes, one with P. *aeruginosa* and one with A. *baumannii*; both were monomicrobial. On-therapy mortality: death occurring during cefepime/zidebactam treatment, regardless of attribution.

^d^Other Gram-negative organisms comprised *S. maltophilia*, *S. marcescens*, and *M. morganii* (one patient each).

^e^Monomicrobial and polymicrobial summary rows include patients across all pathogen categories. The n mono and n poly columns are not applicable to these rows because patients are already grouped by monomicrobial or polymicrobial infection status.

Among patients with isolates tested at the central laboratory (*n* = 72), outcomes were evaluated according to the highest patient-level cefepime/zidebactam MIC (Table 7). Clinical success and microbiologic eradication were both 100.0% among patients with MICs ≤4 mg/L (*n* = 21). At an MIC of 8 mg/L (*n* = 26), clinical success was 80.8% and on-therapy mortality was 19.2%; at 16 mg/L (*n* = 15), clinical success was 86.7% and mortality was 13.3%. Outcomes at higher MICs (32–64 mg/L, *n* = 8; >64 mg/L, *n* = 2) were variable, although interpretation is limited by small sample sizes. Similar findings were observed in the prespecified *P. aeruginosa* subgroup (Table 7).

**Table 7.** Clinical outcomes by cefepime/zidebactam MIC among patients with susceptibility-tested isolates (*n* = 72 patients)^a^

Highest MIC (mg/L)^b,c,d^	Patients (*n*)	Clinical success *n* (%)^e,f^	Microbiologic eradication *n* (%)^e,f^	On-therapy mortality *n* (%)^e^
≤4	21	21 (100.0%)	21 (100.0%)	0 (0.0%)
8	26	21 (80.8%)	23 (88.5%)	5 (19.2%)
16	15	13 (86.7%)	13 (86.7%)	2 (13.3%)
32–64	8	8 (100.0%)	6 (75.0%)	0 (0.0%)
>64	2	1 (50.0%)	2 (100.0%)	1 (50.0%)

^a^Analysis was restricted to 72 of 85 patients with at least one isolate tested for cefepime/zidebactam MIC at the central laboratory. The remaining 13 patients had no central-laboratory MIC data.

^b^For patients with multiple tested isolates, the highest cefepime/zidebactam MIC was used for patient-level analyses, including across organisms in polymicrobial infections.

^c^MIC categories were based on the observed patient-level distribution, with off-scale values (>64 mg/L) analysed separately.

^d^The ≤4 mg/L category included MICs of 0.12, 0.25, 0.5, 1, 2, and 4 mg/L; the 32–64 mg/L category included MICs of 32 and 64 mg/L.

^e^Outcome definitions follow Table 5. Among patients with *P. aeruginosa* infection (*n* = 55), clinical success, microbiological eradication, and on-therapy mortality, respectively, were 100.0%, 100.0%, and 0.0% for MIC ≤4 mg/L (*n* = 13); 80.0%, 88.0%, and 20.0% for MIC 8 mg/L (*n* = 25); 81.8%, 81.8%, and 18.2% for MIC 16 mg/L (*n* = 11); and 100.0%, 83.3%, and 0.0% for MIC 32–64 mg/L (*n* = 6). No patients had MICs >64 mg/L.

^f^Indeterminate outcomes were classified as failures. Three patients had indeterminate microbiological outcomes (MIC 8 mg/L, *n* = 1; MIC 16 mg/L, *n* = 2), and two had indeterminate clinical outcomes (MIC 8 mg/L, *n* = 1; MIC >64 mg/L, *n* = 1).

Among 69 patients with genotyped isolates, outcomes were generally similar regardless of resistance genotype (Table 8). Clinical success was achieved in 87.5% of MBL-positive patients and 92.3% of MBL-negative patients, with microbiologic eradication in 92.9% and 76.9%, respectively. All 14 patients with PBP-3 mutations achieved clinical success, and outcomes were similarly favourable among patients with NDM-positive isolates.

**Table 8.** Clinical outcomes by resistance genotype among patients with available genotypic data (*n* = 69 patients)^a^

Genotype pattern^b^	*n*	Clinical success *n* (%)^c^	Microbiologic eradication *n* (%)^c^	On-therapy mortality *n* (%)
***MBL× PBP-3 mutation status* ^d,e,f^**				
MBL-positive (overall)	56	49 (87.5%)	52 (92.9%)	7 (12.5%)
MBL + PBP-3 mutation	9	9 (100.0%)	9 (100.0%)	1 (11.1%)
MBL, no PBP-3 mutation	47	40 (85.1%)	43 (91.5%)	6 (12.8%)
MBL-negative (overall)	13	12 (92.3%)	10 (76.9%)	1 (7.7%)
MBL− + PBP-3 mutation	5	5 (100.0%)	3 (60.0%)	0 (0.0%)
MBL−, no PBP-3 mutation	8	7 (87.5%)	7 (87.5%)	1 (12.5%)
***Other resistance patterns (*≥*20 patients in positive group)*^g,h^**				
NDM-positive	47	40 (85.1%)	43 (91.5%)	7 (14.9%)
NDM-negative	22	21 (95.5%)	19 (86.4%)	1 (4.5%)
PDC variant present^i^	48	42 (87.5%)	42 (87.5%)	7 (14.6%)
PDC variant absent	21	19 (90.5%)	20 (95.2%)	1 (4.8%)
Porin alterations present^j^	23	22 (95.7%)	21 (91.3%)	2 (8.7%)
Porin alterations absent	46	39 (84.8%)	41 (89.1%)	6 (13.0%)
Quinolone target mutations present^k^	24	22 (91.7%)	21 (87.5%)	2 (8.3%)
Quinolone target mutations absent	45	39 (86.7%)	41 (91.1%)	6 (13.3%)
Aminoglycoside-modifying enzymes present^l^	38	34 (89.5%)	35 (92.1%)	4 (10.5%)
Aminoglycoside-modifying enzymes absent	31	27 (87.1%)	27 (87.1%)	4 (12.9%)

^a^Analysis included 69 of 85 patients with genotypic data available from at least one isolate characterized at the Wockhardt central laboratory. Sixteen patients were excluded: 13 had no isolate received centrally, and three had isolates received but without completed genotypic characterization. One polymicrobial patient contributed two such isolates (*K. pneumoniae* and *P. aeruginosa*); two additional patients each contributed one *P. aeruginosa* isolate.

^b^Patient-level resistance variables were positive when the mechanism was detected in any central-laboratory isolate, including across organisms in polymicrobial infections. Patients with completed characterization but no detected acquired β-lactamase, AmpC variant, or non-enzymatic mechanism were included and classified as negative.

^c^Outcome definitions follow Table 5. Indeterminate outcomes were classified as failures. Three patients had indeterminate microbiological outcomes, two MBL-positive and one MBL-negative; both indeterminate clinical outcomes occurred in MBL-positive, NDM-positive patients. On-therapy mortality: death occurring during cefepime/zidebactam treatment, regardless of attribution.

^d^MBLs included NDM, VIM, IMP, DIM, and the intrinsic L1 enzyme of *S. maltophilia*. NDM predominated.

^e^PBP-3 mutations included amino-acid substitutions or YRIN/YRIK four-amino-acid insertions.

^f^Patients were cross-classified by MBL and PBP-3 mutation status. The four indented subgroups were mutually exclusive and summed to 69. PBP-3 mutations were present in 14 patients: nine MBL-positive and five MBL-negative.

^g^Other resistance patterns were not mutually exclusive and could overlap with the MBL/PBP-3 strata.

^h^Other resistance patterns were limited to those present in at least 20 patients in the genotyped subset. Less common mechanisms are reported descriptively in Table 4.

^i^PDC variants included the *P. aeruginosa* AmpC variants detected in this cohort.

^j^Porin alterations included OprD inactivation, truncation, or substitution in *P. aeruginosa* and analogous OmpK36 alterations in *K. pneumoniae*.

^k^Quinolone target mutations included substitutions in *gyrA*, *parC*, or *parE* associated with fluoroquinolone resistance.

^l^Aminoglycoside-modifying enzymes included acetyltransferases, nucleotidyltransferases, and phosphotransferases detected in this cohort.

Treatment-emergent adverse events were reported in five patients (5.9%; 95% CI 2.5–13.0), including renal dysfunction (*n* = 3), neurological events (*n* = 3), and hepatic enzyme elevations (*n* = 2). Cefepime/zidebactam was withdrawn in one patient after 21 days in the setting of kidney function decline and elevated liver enzymes (Table 1).

## Discussion

In this multi-country compassionate-use analysis, cefepime/zidebactam was associated with high rates of investigator-assessed clinical success and microbiological eradication at the end of treatment in patients with carbapenem-resistant Gram-negative infections and few remaining therapeutic options. The cohort was clinically complex, with more than half of patients receiving care in an intensive care unit at treatment initiation, a substantial burden of comorbidities and acute complications, and extensive prior antimicrobial exposure. Infection types were heterogeneous, with pneumonia or other respiratory infection and bone and joint infection the most common syndromes, and *P. aeruginosa* the predominant pathogen.

A defining feature of this cohort was the predominance of MBL-mediated resistance, particularly among *P. aeruginosa* isolates. MBLs were identified in more than 80% of genotyped isolates, with NDM predominating, and were nearly always accompanied by additional resistance mechanisms, including OXA-type β-lactamases, AmpC variants, and porin or efflux alterations. The frequent co-occurrence of multiple resistance mechanisms likely reflects both the compassionate-use setting, in which cefepime/zidebactam was used on a case-by-case basis for patients with few remaining therapeutic options, and the predominance of patients from India, where NDM-producing pathogens are endemic.^16–18^

Despite the high resistance burden, investigator-assessed clinical success and microbiological eradication among patients with MBL-positive isolates were 87.5% and 92.9%, respectively. PBP-3 modifications were identified in 14 of 69 genotyped patients, all of whom achieved clinical success; nine of these patients also harboured an MBL. Because aztreonam and cefiderocol depend heavily on PBP-3 binding, such alterations have been associated with reduced *in vitro* activity of both agents.^19–22^ In contrast, zidebactam binds PBP-2 with high affinity, providing a mechanistic rationale for preserved activity when MBL production and PBP-3 modification coexist.^7–9^ This distinction is clinically relevant because MBL-producing pathogens remain resistant to most currently available β-lactam/β-lactamase inhibitor combinations.^19–25^ However, the small subgroup sizes, uncontrolled design, and potential influence of concomitant therapy preclude attributing these outcomes to cefepime/zidebactam alone.

Outcomes differed by infection syndrome and baseline severity. Pneumonia/respiratory infection had the lowest investigator-assessed clinical success and highest on-therapy mortality, and all deaths occurred among patients receiving treatment in an intensive care unit. This pattern likely reflects the greater illness severity and treatment complexity of carbapenem-resistant Gram-negative pneumonia, as reported with other newer agents,^26–31^ although cross-study comparisons are limited by differences in case mix, outcome definitions, follow-up, and treatment selection. Outcomes in bone and joint, urinary tract, and central nervous system infections were more favourable but should be interpreted cautiously because these subgroups were small and treatment duration and source control were not standardized.

Clinical success remained high among patients with *P. aeruginosa*, including those with complex resistance profiles, and was similarly favourable among patients with *K. pneumoniae* and *A. baumannii*. The higher on-therapy mortality observed in patients with *P. aeruginosa* likely reflects its predominance among pneumonia cases and greater baseline illness severity rather than reduced cefepime/zidebactam activity against this pathogen. Cefepime/zidebactam MICs ranged from 0.12 to >64 mg/L, whereas comparator MICs were generally high. Outcomes were observed across the measured MIC distribution, including among several patients with MICs of 32–64 mg/L. Preclinical pharmacokinetic/pharmacodynamic studies using stringent efficacy targets, including ≥2 log10 bacterial kill and conservative probability-of-target-attainment thresholds, have supported activity against Gram-negative isolates with MICs up to 64 mg/L.^2,4,6,7,10–14^ The present findings are directionally consistent with those data but should not be interpreted as validating a clinical breakpoint. The small number of patients in the higher MIC strata, absence of measured drug exposures, and potential confounding by disease severity and concomitant therapy preclude definitive conclusions regarding the relationship between MIC and clinical outcome.

Safety findings were favourable, with few reported treatment-emergent adverse events. Cefepime/zidebactam was withdrawn in one patient after 21 days in the setting of kidney function decline and elevated liver enzymes. Although site-based reporting may have undercaptured events, the observed profile was consistent with that of other β-lactams^32–34^ and appeared more favourable than that of polymyxins and aminoglycosides,^35–38^ agents that 42%–45% of patients had received before cefepime/zidebactam.

This analysis has several important limitations. First, it was an uncontrolled retrospective case series; therefore, clinical improvement cannot be attributed to cefepime/zidebactam, and comparisons with historical controls or other therapies would be vulnerable to confounding by indication, temporal changes in care, and differences in patient populations. Concomitant antimicrobial therapy was not consistently captured and may have contributed to the observed outcomes. Second, eligibility for this analysis required at least 4 days of cefepime/zidebactam treatment, thereby excluding patients who died or discontinued treatment earlier and introducing survivor selection that may have inflated estimates of treatment success. Third, clinical success and presumed microbiological eradication were assessed by treating investigators without independent central adjudication or standardized post-treatment evaluation. Fourth, only on-therapy mortality was available; 30-day and 90-day mortality, relapse, and durability of response were not routinely captured. Fifth, compassionate-use decisions, dosing, treatment duration, source control, and supportive care were not standardized, and treatment requests were based on treating-physician assessment rather than prespecified disease-severity criteria. Sixth, only available viable isolates underwent central testing and sequencing, introducing potential microbiological selection bias. Finally, most patients were treated in India, and the resistance epidemiology and care settings may limit generalizability to other regions.

In conclusion, this series provides clinically relevant descriptive evidence for cefepime/zidebactam use in patients with carbapenem-resistant Gram-negative infections, including infections caused by MBL-producing pathogens. Investigator-assessed clinical success and microbiological eradication were high at the end of treatment, and cefepime/zidebactam was generally well tolerated. Prospective controlled studies with standardized outcome assessment, complete capture of concomitant therapy, fixed follow-up, and exposure–response analyses are needed to define comparative effectiveness, safety, and the relationship between MIC and clinical outcome.

## Supplementary Material

dkag324_Supplementary_Data

## Transparency declarations

D.K.D (Dilip Kumar Dubey), R.S. (Rasika Sirsat), A.G. (Abdul Ghafur), R.G. (Raman Gaikwad), V.N. (Vasant Nagvekar), A.M. (Abi Manesh), I.M. (Irfana Mohammed), H.S.L (Harshad Satish Limaye), P.H.S. (Pooja Himanshu Shah), A.M. (Ankit Mittal), N.G. (Nitin Gupta), U.A. (Umang Agrawal), M.S. (Manish Soneja), S.S. (Sowmya Sridharan), S.M. (Surabhi Madan), S.K.M. (Sandeep Kumar Mitra), M.S. (Monalisa Sahu), K.K.T. (Kedar Krishnarao Toraskar), T.S. (Tanu Singhal), A.R.T. (Ashwini Ramakant Tayade), A.S. (Anjely Pulparampil Sebastian), V.R. (Vijil Rahulan), H.S. (Honey Satish Savla), R.N.S (Radhika Narsingdas Sarda), N.B. (Nitin Bansal), S.B.I. (Shalmali B. Inamdar), H.B.K. (Harshith B. Kadnur), S.B.S. (Siddhant B. Shetty), V.D. (Vikas Deswal), T.W. (Timothy William), B.V. (Balaji Veeraraghavan): none to declare.

S.S. (Subramanian Swaminathan) is currently serving as President of the Clinical Infectious Diseases Society of India and has received speaker honoraria from Wockhardt. All other conflicts of interest: none to declare.

P.K.T. (Praveen Kumar Tirlangi) received travel support from Wockhardt Ltd. for attendance at a scientific conference. No other conflicts of interest are declared.

V.R.P. (Vishnu Rao Polati) has received honoraria for lectures from Wockhardt, with payments made directly to him. All other conflicts of interest: none to declare.

R.S. (Rajeev Soman) has received advisory board fees from Pfizer, Glenmark, GSK, Wockhardt, Cipla, bioMérieux, and Emcure.

P.Y.P. (Pratik Y. Patil) has participated in post-marketing surveillance studies for Bharat Serum; has received honoraria from Pfizer, Wockhardt, GSK, and Glenmark; has served on advisory boards for bioMérieux, Glenmark, Pfizer, Wockhardt, and Zydus; and holds Wockhardt stock purchased in a personal capacity.

A.J.S. (Ayesha Jamshed Sunavala) has received honoraria from Pfizer, Glenmark, Wockhardt, BDR Pharma, and Bharat Serums. All other conflicts of interest: none to declare.

N.G. (Neha Gupta) has served as Secretary of the Clinical Infectious Diseases Society (CIDS), India. All other conflicts of interest: none to declare.

S.N. (Suneetha Narreddy) has received honoraria and speakers’ bureau fees from Pfizer, Cipla, GSK, Zydus, and Sanofi; has served on advisory boards for Pfizer, Wockhardt, Cipla, GSK, and Zydus; and has held leadership roles in the Clinical Infectious Disease Society of India, including as Treasurer and Organizing Chair for CIDSCON 2024, as well as membership in the Fungal Infections Study Forum.

E.R.U (Erlinda R. Ulloa) has received honoraria from Sharp Rees-Stealy Hospital. All other conflicts of interest: none to declare.

W.R.M. (William R. Miller) has received a grant from the National Institutes of Health, National Institute of Allergy and Infectious Diseases (NIH/NIAID; Grant No. R21 AI190338); has also received grant support from Merck and Shionogi through his institution; receives royalties from UpToDate as an UpToDate Topic Author; and has received travel support from the Clinical and Laboratory Standards Institute (CLSI). All other conflicts of interest: none to declare.

V.K. (Vidya Krishna) has received speaker fees from Pfizer and Microlabs. All other conflicts of interest: none to declare.

E.A. (Elisabeth Elaine Adderson) has received consulting fees from Shionogi Inc. and honoraria from BMJ Publishing Group, US Advisory Board for educational materials. All other conflicts of interest: none to declare.

S.L.A. (Samuel Lawrence Aitken) has received consulting fees from BioVersys, Shionogi, Innoviva Specialty Therapeutics, Basilea, Roche, Wockhardt, bioMérieux, and Alembic; has received honoraria from Shionogi; serves on the Executive Board of the Society of Infectious Diseases Pharmacists; and is an associate editor for Open Forum Infectious Diseases.

D.v.D. (David van Duin) has received grants or contracts from Merck and the National Institutes of Health (NIH), with payments made to his institution; consulting fees from Pfizer, GSK, Merck, Roche, and Sandoz; has served on advisory boards for Teva and Parexel; and has received an editor stipend from the British Society for Antimicrobial Chemotherapy. All other conflicts of interest: none to declare.

A.S. (Andrew Shorr) has received consulting fees from BioVersys, Shionogi, Pearl Diagnostics, and Basilea. All other conflicts of interest: none to declare.

T.P.L. (Thomas P. Lodise) has received consulting fees from Wockhardt; has received travel support from Wockhardt; and has served on an advisory board for Wockhardt. All other conflicts of interest: none to declare.

## Supplementary data

Appendix S1 and Tables S1 to S4 are available as Supplementary data at *JAC* Online.

## References

[1] Katsarou A, Stathopoulos P, Tzvetanova ID et al Beta-lactam/beta-lactamase inhibitor combination antibiotics under development. Pathogens 2025; 14: 168. 10.3390/pathogens14020168 PMC11858600 40005543

[2] Bhagwat SS, Periasamy H, Takalkar SS et al The novel beta-lactam enhancer zidebactam augments the in vivo pharmacodynamic activity of cefepime in a neutropenic mouse lung Acinetobacter baumannii infection model. Antimicrob Agents Chemother 2019; 63: e02146-18. 10.1128/AAC.02146-18 PMC6437547 30670419

[3] Sader HS, Rhomberg PR, Flamm RK et al WCK 5222 (cefepime/zidebactam) antimicrobial activity tested against Gram-negative organisms producing clinically relevant beta-lactamases. J Antimicrob Chemother 2017; 72: 1696–703. 10.1093/jac/dkx050 28333332

[4] Moya B, Barcelo IM, Cabot G et al In vitro and in vivo activities of beta-lactams in combination with the novel beta-lactam enhancers zidebactam and WCK 5153 against multidrug-resistant metallo-beta-lactamase-producing Klebsiella pneumoniae. Antimicrob Agents Chemother 2019; 63: e00128-19. 10.1128/AAC.00128-19 PMC6496095 30782985

[5] Papp-Wallace KM, Nguyen NQ, Jacobs MR et al Strategic approaches to overcome resistance against Gram-negative pathogens using beta-lactamase inhibitors and beta-lactam enhancers. Activity of three novel diazabicyclooctanes WCK 5153, zidebactam (WCK 5107), and WCK 4234. J Med Chem 2018; 61: 4067–86. 10.1021/acs.jmedchem.8b00091 PMC6131718 29627985

[6] Moya B, Barcelo IM, Bhagwat S et al Potent beta-lactam enhancer activity of zidebactam and WCK 5153 against Acinetobacter baumannii, including carbapenemase-producing clinical isolates. Antimicrob Agents Chemother 2017; 61: e01238-17. 10.1128/AAC.01238-17 PMC5655067 28848013

[7] Moya B, Barcelo IM, Bhagwat S et al WCK 5107 (zidebactam) and WCK 5153 are novel inhibitors of PBP2 showing potent “beta-lactam enhancer” activity against Pseudomonas aeruginosa, including multidrug-resistant metallo-beta-lactamase-producing high-risk clones. Antimicrob Agents Chemother 2017; 61: e02529-16. 10.1128/AAC.02529-16 PMC5444176 28289035

[8] Moya B, Bhagwat S, Cabot G et al Effective inhibition of PBPs by cefepime and zidebactam in the presence of VIM-1 drives potent bactericidal activity against MBL-expressing *Pseudomonas aeruginosa*. J Antimicrob Chemother 2020; 75: 1474–8. 10.1093/jac/dkaa036 32083659

[9] Hujer AM, Marshall SH, Mack AR et al Transcending the challenge of evolving resistance mechanisms in *Pseudomonas aeruginosa* through beta-lactam-enhancer-mechanism-based cefepime/zidebactam. mBio 2023; 14: e0111823. 10.1128/mbio.01118-23 PMC10746216 37889005

[10] Sader HS, Mendes RE, Duncan LR et al Antimicrobial activity of cefepime/zidebactam (WCK 5222), a beta-lactam/beta-lactam enhancer combination, against clinical isolates of Gram-negative bacteria collected worldwide (2018-19). J Antimicrob Chemother 2022; 77: 2642–9. 10.1093/jac/dkac233 35897129

[11] Monogue ML, Tabor-Rennie J, Abdelraouf K et al In vivo efficacy of WCK 5222 (cefepime-zidebactam) against multidrug-resistant Pseudomonas aeruginosa in the neutropenic murine thigh infection model. Antimicrob Agents Chemother 2019; 63: 10.1128/aac.00233-19. 10.1128/AAC.00233-19 PMC6591597 31235557

[12] Almarzoky Abuhussain SS, Avery LM, Abdelraouf K et al In vivo efficacy of humanized WCK 5222 (cefepime-zidebactam) exposures against carbapenem-resistant Acinetobacter baumannii in the neutropenic thigh model. Antimicrob Agents Chemother 2019; 63: e01931-18. 10.1128/AAC.01931-18 PMC6325183 30373797

[13] Lasko MJ, Abdelraouf K, Nicolau DP. Comparative in vivo activity of human-simulated plasma and epithelial lining fluid exposures of WCK 5222 (cefepime/zidebactam) against KPC- and OXA-48-like-producing *Klebsiella pneumoniae* in the neutropenic murine pneumonia model. J Antimicrob Chemother 2021; 76: 2310–6. 10.1093/jac/dkab183 34096601

[14] Kidd JM, Abdelraouf K, Nicolau DP. Efficacy of human-simulated bronchopulmonary exposures of cefepime, zidebactam and the combination (WCK 5222) against MDR Pseudomonas aeruginosa in a neutropenic murine pneumonia model. J Antimicrob Chemother 2020; 75: 149–55. 10.1093/jac/dkz414 31641765

[15] CLSI . *Methods for Dilution Antimicrobial Susceptibility Tests for Bacteria That Grow Aerobically—Twelfth Edition: M07*. 2024.

[16] Dadashi M, Yaslianifard S, Hajikhani B et al Frequency distribution, genotypes and prevalent sequence types of New Delhi metallo-beta-lactamase-producing Escherichia coli among clinical isolates around the world: a review. J Glob Antimicrob Resist 2019; 19: 284–93. 10.1016/j.jgar.2019.06.008 31212107

[17] Rahman M, Shukla SK, Prasad KN et al Prevalence and molecular characterisation of New Delhi metallo-beta-lactamases NDM-1, NDM-5, NDM-6 and NDM-7 in multidrug-resistant Enterobacteriaceae from India. Int J Antimicrob Agents 2014; 44: 30–7. 10.1016/j.ijantimicag.2014.03.003 24831713

[18] Deshpande P, Rodrigues C, Shetty A et al New Delhi Metallo-beta lactamase (NDM-1) in Enterobacteriaceae: treatment options with carbapenems compromised. J Assoc Physicians India 2010; 58: 147–9. 20848811

[19] Le Terrier C, Nordmann P, Buchs C et al Effect of modification of penicillin-binding protein 3 on susceptibility to ceftazidime-avibactam, imipenem-relebactam, meropenem-vaborbactam, aztreonam-avibactam, cefepime-taniborbactam, and cefiderocol of Escherichia coli strains producing broad-spectrum beta-lactamases. Antimicrob Agents Chemother 2024; 68: e0154823. 10.1128/aac.01548-23 PMC10989025 38415988

[20] Le Terrier C, Nordmann P, Freret C et al Impact of acquired broad spectrum beta-lactamases on susceptibility to novel combinations made of beta-lactams (aztreonam, cefepime, meropenem, and imipenem) and novel beta-lactamase inhibitors in Escherichia coli and Pseudomonas aeruginosa. Antimicrob Agents Chemother 2023; 67: e0033923. 10.1128/aac.00339-23 PMC10353362 37255469

[21] Periasamy H, Joshi P, Palwe S et al High prevalence of Escherichia coli clinical isolates in India harbouring four amino acid inserts in PBP3 adversely impacting activity of aztreonam/avibactam. J Antimicrob Chemother 2020; 75: 1650–1. 10.1093/jac/dkaa021 32040179

[22] Poirel L, Ortiz de la Rosa JM, Sadek M et al Impact of acquired broad-spectrum beta-lactamases on susceptibility to cefiderocol and newly developed beta-lactam/beta-lactamase inhibitor combinations in Escherichia coli and Pseudomonas aeruginosa. Antimicrob Agents Chemother 2022; 66: e0003922. 10.1128/aac.00039-22 PMC9017383 35315685

[23] Bush K, Bradford PA. Beta-lactams and beta-lactamase inhibitors: an overview. Cold Spring Harb Perspect Med 2016; 6: a025247. 10.1101/cshperspect.a025247 PMC4968164 27329032

[24] Tamma PD, Heil EL, Justo JA et al Infectious Diseases Society of America 2024 guidance on the treatment of antimicrobial-resistant Gram-negative infections. Clin Infect Dis 2024; 10.1093/cid/ciae403 39108079

[25] Doi Y . Treatment options for carbapenem-resistant Gram-negative bacterial infections. Clin Infect Dis 2019; 69: S565–75. 10.1093/cid/ciz830 PMC6853760 31724043

[26] Garcia-Carnero L, Abelenda-Alonso G, Santos-Puig M et al Cefiderocol targeted treatment for multidrug-resistant Gram-negative infections: an observational cohort study. Antibiotics (Basel) 2026; 15: 416. 10.3390/antibiotics15040416 PMC13113607 42041379

[27] Marino A, Venanzi Rullo E, Russotto Y et al Real-world effectiveness and safety of cefiderocol in critically ill patients with MDR Gram-negative infections: results from a retrospective study. Biomed Rep 2025; 23: 156. 10.3892/br.2025.2034 PMC12312293 40756900

[28] Risco-Risco C, Henriquez-Camacho C, Herrera-Rueda M et al Cefiderocol versus best available therapy in the treatment of critically ill patients with severe infections due to resistant Gram-negative bacteria: a systematic review and meta-analysis. Antibiotics (Basel) 2024; 13: 1048. 10.3390/antibiotics13111048 PMC11591197 39596742

[29] Lodise TP, Min J, Nathanson BH et al Comparison of early treatment with ceftolozane/tazobactam versus polymyxin-based therapy of pneumonia due to MDR Pseudomonas aeruginosa (PUMA). Antimicrob Agents Chemother 2025; 69: e0056925. 10.1128/aac.00569-25 PMC12587572 40965589

[30] Lodise TP, Obi EN, Watanabe AH et al Comparative evaluation of early treatment with ceftolozane/tazobactam versus ceftazidime/avibactam for non-COVID-19 patients with pneumonia due to multidrug-resistant Pseudomonas aeruginosa. J Antimicrob Chemother 2024; 79: 2954–64. 10.1093/jac/dkae313 PMC11531822 39258877

[31] Hareza DA, Cosgrove SE, Bonomo RA et al Clinical outcomes and emergence of resistance of Pseudomonas aeruginosa infections treated with ceftolozane-tazobactam versus ceftazidime-avibactam. Antimicrob Agents Chemother 2024; 68: e0090724. 10.1128/aac.00907-24 PMC11459953 39230311

[32] Baldwin CM, Lyseng-Williamson KA, Keam SJ. Meropenem: a review of its use in the treatment of serious bacterial infections. Drugs 2008; 68: 803–38. 10.2165/00003495-200868060-00006 18416587

[33] Meropenem for Injection . U.S. Food and Drug Administration. Revised 2024. Accessed March 24, 2026. https://www.accessdata.fda.gov/drugsatfda_docs/label/2024/202106s009lbl.pdf.

[34] Lagace-Wiens P, Rubinstein E. Adverse reactions to beta-lactam antimicrobials. Expert Opin Drug Saf 2012; 11: 381–99. 10.1517/14740338.2012.643866 22220627

[35] Krause KM, Serio AW, Kane TR et al Aminoglycosides: an overview. Cold Spring Harb Perspect Med 2016; 6: a027029. 10.1101/cshperspect.a027029 PMC4888811 27252397

[36] Kelesidis T, Falagas ME. The safety of polymyxin antibiotics. Expert Opin Drug Saf 2015; 14: 1687–701. 10.1517/14740338.2015.1088520 PMC8082492 26365594

[37] Vidal L, Gafter-Gvili A, Borok S et al Efficacy and safety of aminoglycoside monotherapy: systematic review and meta-analysis of randomized controlled trials. J Antimicrob Chemother 2007; 60: 247–57. 10.1093/jac/dkm193 17562680

[38] Falagas ME, Kasiakou SK. Toxicity of polymyxins: a systematic review of the evidence from old and recent studies. Crit Care 2006; 10: R27. 10.1186/cc3995 PMC1550802 16507149

