# Supplementary material for: Real-world experience with cefepime/zidebactam under compassionate use
Source: J Antimicrob Chemother. 2026 Nov 10;81(12):dkag324. doi: 10.1093/jac/dkag324 (PMC13748750; doi:10.1093/jac/dkag324)
Supplement: Supplementary file 1 [file dkag324_supplementary_data.docx]

Real-World Experience with Cefepime/zidebactam Under Compassionate Use

**Supplementary Table 1**. MIC Distribution of Cefepime/zidebactam (n = 77 isolates)

| **MIC (mg/L)** | **Number of isolates, n (%)** | **Cumulative n (%)** |
| --- | --- | --- |
| 0.12 | 1 (1.3) | 1 (1.3) |
| 0.25 | 1 (1.3) | 2 (2.6) |
| 0.5 | 2 (2.6) | 4 (5.2) |
| 1 | 2 (2.6) | 6 (7.8) |
| 2 | 3 (3.9) | 9 (11.7) |
| 4 | 15 (19.5) | 24 (31.2) |
| 8 | 28 (36.4) | 52 (67.5) |
| 16 | 15 (19.5) | 67 (87.0) |
| 32 | 6 (7.8) | 73 (94.8) |
| 64 | 2 (2.6) | 75 (97.4) |
| >64 | 2 (2.6) | 77 (100.0) |

Footnotes:

a. MIC testing was performed by reference broth microdilution according to CLSI M07 methods on available viable isolates received by the Wockhardt central reference laboratory.

b. Multiple isolates per patient were included where available; therefore, isolate-level analyses may not correspond directly to patient-level counts reported elsewhere.

c. MIC values are reported as doubling dilutions (mg/L); values exceeding the upper assay limit are reported as greater than the highest concentration tested (>64 mg/L).

d. Percentages are calculated using the total number of tested isolates (n = 77) as the denominator.

e. Cumulative percentages represent the proportion of isolates at or below each MIC value.

**Supplementary Table 2**. Cefepime/zidebactam MIC Distribution by Pathogen Among Tested Isolates (n = 77 isolates)

| **Pathogen** | **n (isolates)** | **MIC range (mg/L)** | **MIC_50_** | **MIC_90_** | **>64 mg/L, n (%)** |
| --- | --- | --- | --- | --- | --- |
| *P. aeruginosa* | 55 | 1–64 | 8 | 16 | 0 (0.0%) |
| *K. pneumoniae* | 8 | 0.25–32 | 2 | 32 | 0 (0.0%) |
| *A. baumannii* | 9 | 8–>64 | 16 | >64 | 2 (22.2%) |
| *E. coli* | 2 | 0.12–0.5 | NA | NA | 0 (0.0%) |
| Other Gram-negatives | 3 | 4–8 | NA | NA | 0 (0.0%) |

Footnotes:

a. Isolate-level summary based on 77 available viable isolates received and tested by the Wockhardt central reference laboratory using reference broth microdilution methods.

b. For pathogen groups with n < 6 isolates (*E. coli* and Other Gram-negatives), MIC_50_ and MIC_90_ are reported as “NA” given the limited number of observations.

c. “>64 mg/L” column reports the number (%) of isolates with cefepime/zidebactam MIC explicitly >64 mg/L (off-scale).

d. “Other Gram-negatives” includes *Stenotrophomonas maltophilia*, *Serratia marcescens*, and *Morganella morganii* (one isolate each).

**Supplementary Table 3**. Patient-Level Distribution of Resistance Mechanisms Among Patients with Available Genotypic Data (n = 69 patients)

| **Pathogen** | **n (patients)** | **MBL**  **n (%)** | **OXA-type**  **n (%)** | **ESBL/other**  **n (%)** | **AmpC variants**  **n (%)** | **Non-enzymatic n (%)** |
| --- | --- | --- | --- | --- | --- | --- |
| *P. aeruginosa* | 52 | 43 (82.7%) | 48 (92.3%) | 1 (1.9%) | 49 (94.2%) | 46 (88.5%) |
| *K. pneumoniae* | 7 | 5 (71.4%) | 6 (85.7%) | 6 (85.7%) | 1 (14.3%) | 5 (71.4%) |
| *A. baumannii* | 9 | 7 (77.8%) | 9 (100.0%) | 1 (11.1%) | 9 (100.0%) | 9 (100.0%) |
| *E. coli* | 2 | 2 (100.0%) | 2 (100.0%) | 1 (50.0%) | 1 (50.0%) | 2 (100.0%) |
| Other Gram-negatives | 3 | 2 (66.7%) | 1 (33.3%) | 1 (33.3%) | 2 (66.7%) | 2 (66.7%) |

Footnotes:

a. This analysis includes 69 of 85 patients with available genotypic data from at least one isolate characterized at the Wockhardt central reference laboratory. The 16 excluded patients comprised 13 without an isolate received by the central laboratory and 3 whose isolates had not completed genotypic characterization.

b. For each pathogen group, the denominator reflects the number of unique patients with at least one characterized isolate of that organism. Resistance-mechanism indicators were aggregated to the patient level based on the presence of the mechanism in any available isolate of the same organism.

c. Resistance mechanism categories are not mutually exclusive; patients can carry multiple resistance mechanisms simultaneously.

d. Resistance-mechanism categories and definitions follow **Table 4** of the main manuscript.

**Supplementary Table 4**. Clinical Outcomes by Baseline Illness Severity and Host Status (n = 85)

| **Subgroup** | **n** | **Clinical success at the end of therapy**  **n (%)** | **Microbiologic eradication at the end of therapy**  **n (%)** | **On-therapy mortality n (%)** |
| --- | --- | --- | --- | --- |
| **Critical-illness status at initiation** | | | | |
| ICU at initiation | 49 | 40 (81.6%) | 43 (87.8%) | 9 (18.4%) |
| Not in ICU at initiation | 36 | 35 (97.2%) | 33 (91.7%) | 0 (0.0%) |
| **Sepsis-spectrum complications during episode** | | | | |
| Sepsis / septic shock / MODS | 32 | 25 (78.1%) | 27 (84.4%) | 8 (25.0%) |
| No sepsis / septic shock / MODS | 53 | 50 (94.3%) | 49 (92.5%) | 1 (1.9%) |
| **Immunocompromised / transplant-related status** | | | | |
| Immunocompromised / transplant-related | 27 | 22 (81.5%) | 25 (92.6%) | 5 (18.5%) |
| Not immunocompromised | 58 | 53 (91.4%) | 51 (87.9%) | 4 (6.9%) |

Footnotes:

a. Patient-level analysis of the full cohort (n = 85). The three subgroup classifications were intensive care unit status at cefepime/zidebactam initiation, presence of sepsis-spectrum complications during the infection episode, and immunocompromised or transplant-related status.

b. Clinical success and microbiological eradication were assessed by the treating investigator at each site and were not independently adjudicated. Indeterminate outcomes were classified as failures. All five patients with indeterminate outcomes (three microbiological and two clinical) were receiving care in an intensive care unit at cefepime/zidebactam initiation and had sepsis-spectrum complications; three were immunocompromised or transplant-related.

c. All nine on-therapy deaths occurred among patients receiving care in an intensive care unit at treatment initiation; eight had sepsis-spectrum complications and five were immunocompromised or transplant-related.

Statistical note: Results are descriptive; no formal hypothesis testing was performed.
